# From Mechanical Perturbation to Pathological Locking: Temporal–Mechanical Evolution of the Postsynaptic Density After Traumatic Brain Injury

**DOI:** 10.1002/cns.71260

**Published:** 2026-11-06

**Authors:** Yibin Jia, Zixuan Cao, Hanjiang Xue, Zhebin Feng, Zhiguo Ma, Zhicheng Tian, Xin Li, Peng Luo

**Affiliations:** ^1^ Department of Neurosurgery, Xijing Hospital Fourth Military Medical University Xi'an China; ^2^ Department of Neurosurgery PLA 942 Hospital Yinchuan China; ^3^ Department of Anesthesiology, Xijing Hospital Fourth Military Medical University Xi'an China

**Keywords:** mechanobiology, mechanosensation, postsynaptic density, traumatic brain injury

## Abstract

**Background:**

Traumatic brain injury (TBI) induces persistent cognitive deficits, yet conventional biochemical and structural models often fail to explain synaptic signal collapse in the absence of overt tissue rupture.

**Results:**

Here, we present a hypothesis‐generating mechanobiological framework that positions postsynaptic density (PSD)—a multivalent protein condensate governed by liquid–liquid phase separation (LLPS)—as a primary locus of synaptic mechanovulnerability. We argue that TBI‐induced synaptic pathology is not merely a quantitative loss of molecular components, but a progressive, staged material state transition of the PSD network. This pathological evolution initiates with hyperacute mechanical deconstruction, leading to physical scaffold decoupling and nanoscale receptor misalignment. It then advances through an acute mechano‐metabolic crisis characterized by enzymatic scaffold cleavage and impaired re‐condensation, ultimately culminating in a chronically locked, low‐dynamic “solidified” state. Furthermore, post‐injury extracellular matrix stiffening and the mechanical exposure of cryptic epitopes trigger aberrant microglial pruning via mechanosensors such as Piezo1, permanently dismantling neural circuits. Because the supporting evidence is heterogeneous, we explicitly grade each element of this model as established directly in TBI, supported in non‐TBI synaptic systems, extrapolated from reconstituted condensates, or hypothetical.

**Conclusions:**

By bridging macroscopic biomechanics with nanoscale molecular pathology, this review highlights a critical conceptual gap and proposes “mechano‐therapeutics” as a hypothesis‐generating concept rather than an established treatment avenue. We suggest that strategies targeting the physical properties of the synapse, such as stabilizing scaffold oligomerization, modulating mechanotransduction pathways, and restoring physiological tissue stiffness, are candidate directions whose feasibility, delivery, cell‐type specificity, timing, and safety must first be established in preclinical models before any clinical translation to rescue synaptic homeostasis and cognitive function after TBI.

## Graphical abstract

The postsynaptic density (PSD) may convert traumatic mechanical loading into persistent synaptic dysfunction. This review proposes a temporal–mechanical model in which PSD injury progresses from hyperacute mechanical deconstruction (Phase I) through an acute‐to‐subacute metabolic–mechanical crisis (Phase II) to chronic low‐dynamic pathological locking (Phase III). The model is hypothesis‐generating: Phase II changes are observed in TBI models, whereas the Phase I and Phase III states are partly inferred and predictive, as indicated by the evidence tags.

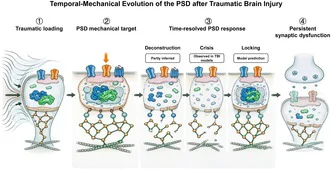

**Abbreviations**
AD: Alzheimer's disease
AMPAR: α‐amino‐3‐hydroxy‐5‐methyl‐4‐isoxazolepropionic acid receptor
APP: amyloid precursor protein
AQP4: aquaporin‐4
Aβ: amyloid‐β
CaMKII: Ca^2+^/calmodulin‐dependent protein kinase II
CCI: controlled cortical impact
CTE: chronic traumatic encephalopathy
ECM: extracellular matrix
GluA1 (GluR1): AMPA receptor subunit 1
GluN (NR): NMDA receptor subunit
GSK3β: glycogen synthase kinase 3β
LINC: linker of nucleoskeleton and cytoskeleton
LLPS: liquid–liquid phase separation
LTD: long‐term depression
LTP: long‐term potentiation
mGluR1: metabotropic glutamate receptor 1
mTBI: mild traumatic brain injury
NMDAR: N‐methyl‐D‐aspartate receptor
PBBI: penetrating ballistic‐like brain injury
PD: Parkinson's disease
PSD: postsynaptic density
rmTBI: repetitive mild traumatic brain injury
ROCK: Rho‐associated coiled‐coil‐containing protein kinase
TARP: transmembrane AMPA receptor regulatory protein
TBI: traumatic brain injury
TNI: traumatic neuronal injury (in vitro)

## 1 Introduction

Traumatic brain injury (TBI) is a global health burden, affecting approximately 69 million individuals annually and reaching an adult lifetime prevalence of up to 29% [1, 2, 3]. Even initially asymptomatic or mild exposures can be followed by cumulative damage associated with Alzheimer's disease (AD), Parkinson's disease (PD), and chronic traumatic encephalopathy (CTE) [4, 5]. The gap between apparently normal clinical imaging and later cognitive decline suggests that post‐traumatic impairment may involve occult microscale alterations of synaptic architecture.

Although cellular mechanotransduction is a fundamental biological regulator [6, 7], TBI research has traditionally emphasized macro‐scale pathology, including neuronal death, axonal shearing, and secondary neuroinflammatory cascades [8, 9, 10, 11]. Frameworks centered on ionic flux and metabolic reprogramming [11, 12, 13, 14] do not fully explain synaptic signaling failure without overt necrosis or structural disruption. This gap reflects insufficient integration of brain tissue mechanics into TBI models, as biochemical approaches alone cannot capture how external forces are physically transmitted into pathophysiological cascades [7, 8, 15, 16]. Consequently, fine‐scale synaptic damage remains under‐recognized.

The postsynaptic density (PSD) is a protein‐dense assembly at excitatory postsynaptic sites that supports synaptic stability and memory [17, 18, 19]. Through core scaffold proteins, including PSD‐95, Shank, and SynGAP1, the PSD forms biomolecular condensates that can couple mechanical responsiveness with secondary injury signaling [20, 21, 22, 23, 24, 25, 26]. We propose a temporal–mechanical perspective in which the PSD acts as a mechano‐signaling hub that converts mechanical perturbation into biochemical dysfunction [18, 27, 28]. Defining the mechanical responses of PSD components may help identify early synaptic injury in mild TBI and guide strategies to preserve neuroplasticity [27, 29, 30].

## 2 PSD—From Protein Complex to Mechanical Material

### 2.1 Hierarchical Organization of the PSD

The postsynaptic density (PSD) is a protein‐dense, membrane‐less assembly at excitatory postsynaptic sites, hierarchically organized to align postsynaptic receptors with presynaptic release sites [20, 21, 22, 31]. As a biomolecular condensate driven by liquid–liquid phase separation (LLPS), it clusters signaling machinery within a permeable molecular network [24, 25, 26, 32, 33]. At the receptor‐facing layer, N‐methyl‐D‐aspartate receptors (NMDARs) and α‐amino‐3‐hydroxy‐5‐methyl‐4‐isoxazolepropionic acid receptors (AMPARs) mediate calcium influx and fast transmission, and their density determines synaptic strength [21, 34, 35]. This interface is enriched with CaMKII, which supports activity‐dependent synaptic efficacy [21, 36], and Preso, which stabilizes mGluR1–Homer1 complexes involved in glutamatergic neurotransmission [37]. Beneath this layer, PSD‐95 anchors glutamate receptors into functional “slots” through its PDZ domains [19, 22, 38, 39]. SynGAP1 acts both as a Ras‐GAP and as a structural competitor within PSD‐95‐associated space; its displacement supports receptor remodeling, whereas its loss is associated with post‐TBI hyperexcitability [24, 40, 41, 42]. SAPAP/GKAP links the PSD‐95 layer to the deeper Shank scaffold [38, 43, 44]. Its flexible N‐terminus may buffer minor compressive forces through a phosphorylation‐sensitive interface [43, 45], whereas SAPAP also modulates local protein translation through NMDAR‐related signaling [46]. At the basal layer, Shank proteins form SAM‐domain oligomeric networks that tether the PSD to actin [33, 47, 48]. Shank3–SAPAP interactions provide local flexibility under mechanical load [49], and Homer proteins connect the scaffold to endoplasmic reticulum calcium stores and actin bundling [33, 50, 51]. Together, these interactions support the structural and mechanical integrity required for coordinated synaptic transmission [17, 18, 19].

### 2.2 Mechanical Properties and Vulnerability of the PSD

The PSD is therefore not only a protein complex but also a viscoelastic condensate with material‐like properties. Its membrane‐less architecture depends on multivalent interactions that preserve topological integrity while allowing component exchange [25, 26, 32, 33]. This organization maintains signaling fidelity by anchoring receptors through the PSD‐95/SAPAP axis within the PSD core [19, 25, 28, 43, 52].

PSD condensates exhibit dual‐mode mechanical relaxation: short‐term exponential relaxation provides initial stiffness, whereas long‐term power‐law relaxation supports slower network remodeling [25]. Mechanical homeostasis also depends on coexistence between an immobile Shank–SAPAP‐rich fraction and a mobile fraction that enables molecular exchange [25, 38, 47, 53]. This solid–liquid coexistence allows the PSD to dissipate physiological loads while maintaining signaling robustness despite rapid protein turnover [45, 47].

The PSD elastic modulus is regulated by internal assembly density, with Shank3 SAM‐domain oligomerization acting as a key determinant of cross‐linking density [47, 48]. A normal oligomeric network gives the PSD soft glass‐like properties that balance stiffness and flexibility [47]. Under excessive loading, however, the physical interactions maintaining network toughness may fail [4, 54]. Once external load exceeds this threshold, the PSD may undergo a mechanically induced phase transition from an ordered condensate into a diffuse or fragmented structure, disrupting local mechanical homeostasis and linking mechanical perturbation to synaptic dysfunction [4, 22, 47, 55] (Figure 1).

**FIGURE 1:**
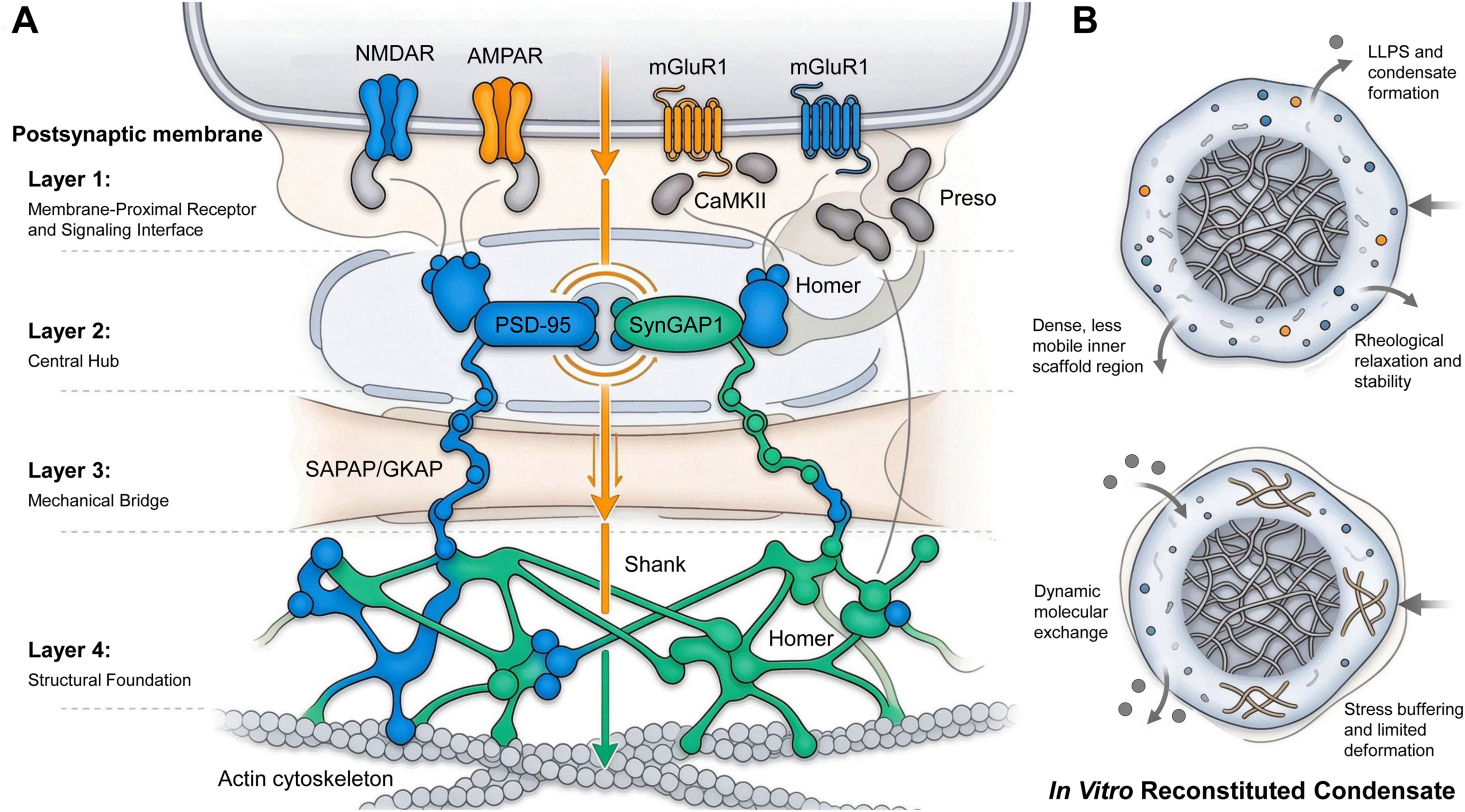
Layered organization and material properties of the postsynaptic density (PSD). (A) Schematic of the vertically organized PSD beneath the postsynaptic membrane of an excitatory synapse. The membrane‐proximal layer (Layer 1) contains receptors and signaling components, including NMDARs, AMPARs, mGluR1, CaMKII, and Preso. PSD‐95 and SynGAP1 form a central hub (Layer 2) for receptor anchoring and local signaling, SAPAP/GKAP acts as a mechanical bridge (Layer 3) to the deeper Shank/Homer scaffold and the Actin cytoskeleton (Layer 4), together forming a receptor‐to‐cytoskeleton load‐bearing axis. The four‐layer grouping is a simplified schematic based on reconstituted and structural studies [18, 20, 21, 22, 31, 32, 33, 43, 47] and does not imply a settled mechanical hierarchy in vivo. (B) The PSD illustrated as a membrane‐less condensate in which a dense, less mobile inner scaffold coexists with a dynamic, exchangeable fraction, supporting deformation, stress buffering, molecular turnover, and local rearrangement under force. Panel (B) depicts material properties demonstrated in reconstituted condensates in vitro [25, 33, 47]; equivalent rheology has not been demonstrated in injured brain tissue and is shown as a model. Evidence key: Solid elements denote pathways established in the cited studies; dashed elements denote inferred or hypothetical features.

An important evidential boundary applies to this material description. The condensate properties summarized above derive largely from reconstituted PSD assemblies and purified‐protein systems studied in vitro. Living synapses differ from these systems in protein concentration and molecular crowding, post‐translational modification states, the presence of membranes, ATP availability, pH, temperature, a viscoelastic cytoskeleton, and ongoing neuronal activity, each of which can shift phase behavior. We therefore use “condensate” to denote a membrane‐less, multivalent assembly and do not assume that a liquid phase has been demonstrated in injured brain tissue unless direct in vivo evidence is cited; the material states discussed below should be read as model predictions to be tested in situ.

### 2.3 Operational Definitions and Multi‐Scale Mechanical Estimates

To use mechanical terms precisely, we define them operationally. Strain is the fractional deformation of tissue, and strain rate is its time derivative; “high strain rates” in experimental TBI models typically refer to loading on the order of 10–100 s^−1^, whereas physiological rates are far lower [56, 57]. “Critical stress” denotes the applied stress at which a defined structure loses integrity; for the PSD this threshold has not been measured and is treated here as an unknown to be determined experimentally. “Tissue stiffness” refers to the elastic or shear modulus, which for brain tissue is on the order of 10^2^–10^3^ Pa, roughly two to three orders of magnitude below rigid culture substrates [58, 59]. “Membrane tension” is the in‐plane force per unit length of the bilayer, typically in the pN/μm range [60]. “Mechanical locking” denotes a state in which component exchange and conformational rearrangement are kinetically arrested, not a defined stress value.

A simple order‐of‐magnitude analysis illustrates how a macroscopic impact could reach individual PSD components. Concussion‐level head motions correspond to peak linear accelerations of roughly 10^2^–10^3^ m s^−2^ and rotational accelerations of 10^3^–10^4^ rad s^−2^ [2, 61]. Because brain tissue is soft, with a shear modulus on the order of 10^3^ Pa [58], such motions generate tissue‐level stresses of approximately 10^2^–10^3^ Pa at strains of several percent to tens of percent [2, 56, 58]. Stress concentrates at interfaces with mismatched elastic moduli, so a synaptic junction of roughly 0.05–0.1 μm^2^ may transiently carry a total force on the order of 10^−11^–10^−10^ N (approximately 10–100 pN). Distributed across the hundreds to thousands of multivalent scaffold interactions within one PSD, this corresponds to sub‐piconewton to few‐piconewton increments per interaction, which are comparable to thermal energies (kBT ≈ 4 pN·nm) and well below covalent bond strengths or typical protein domain unfolding forces of tens of piconewtons [62, 63]. This chain of estimates offers a physical rationale for why non‐covalent, multivalent PSD interactions can fail under injurious loading while primary protein structures remain intact, and it identifies the per‐synapse force scale (tens of piconewtons) as a key quantity for direct measurement.

Where quantitative thresholds are not established, we state this explicitly. The strain or stress at which PSD condensates lose integrity, the membrane tension required to open synaptic Piezo1 channels in situ, and the stiffness threshold that would operationally define “mechanical locking” are currently unknown. We propose that combining calibrated in vitro loading with finite element brain models and in situ force measurements can convert these unknowns into measured values [61, 62, 64, 65].

### 2.4 Evidence Grading and Terminology

Because the evidence underlying this framework is heterogeneous, we assign each central mechanistic claim to one of four evidence levels: (A) established directly in TBI models or human TBI tissue; (B) supported in non‐TBI synaptic or cellular systems; (C) extrapolated from in vitro or reconstituted condensate experiments; and (D) hypothetical model prediction. Table 1 applies this taxonomy to the key claims of this review, including the proposed low‐dynamic “locked” PSD state, receptor mechanogating, SynGAP1 slot competition, extracellular matrix mechanical memory, and microglial pruning. Regarding terminology, we adopt “low‐dynamic pathological locking” as the preferred term for the chronic PSD state; “solidified” and “kinetic freezing” are used only as descriptive synonyms for the same proposed state. This state remains a level‐D prediction: it is inferred from chronic synaptic dysfunction and condensate physics but has not been directly demonstrated in injured brain tissue.

**TABLE 1:** Evidence grading of the central claims of the temporal–mechanical model.

Key claim	Evidence level	Basis	Key references
TBI causes acute loss and fragmentation of PSD scaffold proteins (PSD‐95, Shank3, SynGAP1)	A	Consistent across multiple TBI models and species within hours to days	[22, 38, 54, 66, 67, 68, 69]
Hyperacute nanoscale receptor misalignment precedes protein loss	D	Predicted from condensate mechanics; not yet measured in injured brain	[25, 47]
NMDARs are mechanically gated by membrane stretch	B	Demonstrated in recombinant and cellular systems; not yet shown in TBI tissue	[70]
Piezo1 is an entry point for traumatic mechanical loading	B	Channel mechanism established; TBI role supported indirectly	[71, 72, 73, 74, 75]
CaMKII‐driven SynGAP1 desorption and PSD‐95 slot competition	B/C	SynGAP1 structural role in non‐TBI synapses; competition shown in reconstituted condensates	[41, 42, 52]
The PSD behaves as a phase‐separated condensate with dual‐mode mechanical relaxation	C	Reconstituted PSD condensates in vitro	[18, 25, 47, 52]
Post‐injury ATP depletion impairs PSD re‐condensation	C/D	ATP dependence of condensate dynamics in vitro; post‐TBI ATP depletion is established (A), but the causal link is untested	[14, 25, 76]
ECM stiffening imposes chronic mechanical memory on synapses	B/D	ECM remodeling after TBI (A); stiffness–synapse coupling shown in non‐TBI systems	[30, 59, 77, 78, 79]
Mechanically exposed epitopes gate microglial pruning of damaged PSDs	B/D	Complement‐mediated pruning established; mechanical gating inferred	[22, 80, 81]
Chronic low‐dynamic pathological locking (“kinetic freezing”) of the PSD	D	Model prediction from condensate physics and chronic dysfunction; no direct in vivo measurement	[47, 55]

*Note:* Level A, established directly in TBI models or human TBI tissue; Level B, supported in non‐TBI synaptic or cellular systems; Level C, extrapolated from in vitro or reconstituted condensate experiments; Level D, hypothetical model prediction.

## 3 The PSD Participates in Mechanotransduction Pathways

Traumatic brain injury (TBI) transmits physical forces across the brain's macro‐ and microscopic architecture [4]. PSD injury involves three linked processes: mechanosensitive channels generate early calcium transients [70, 71, 82]; postsynaptic scaffold proteins undergo conformational change, protein–protein dissociation, and phase transition [15, 22, 47, 55]; and downstream signaling pathways amplify PSD network destabilization [8, 14, 42, 82]. PSD condensates can buffer moderate physiological loading, but excessive load disrupts the scaffold framework and converts transient deformation into molecular disconnection [47, 55].

### 3.1 Macro‐to‐Micro Stress Convergence

The mechanotransduction chain in TBI is shaped by coupling among neurons, the extracellular matrix (ECM), cytoskeleton, and integrins. Together, these structures form a continuous network spanning intra‐ and extracellular spaces [7, 83]. Actin sustains tensile loads, whereas microtubules resist compression, creating a stress‐integration system [63, 84]. Integrins connect the cytoskeleton to the ECM and nuclear envelope through the LINC complex, forming a mechanical axis from the extracellular environment to intracellular structures [83, 85].

TBI‐induced stretching disrupts synaptic cleft homeostasis and strains transmembrane adhesion networks [15, 22]. Injury also alters the physical properties of brain ECM, which is normally low in stiffness because of limited fibrous protein content [15, 86]. Stiffness changes are sensed by mechanosensitive structures such as Talin1, whose R1R2 interface participates in rigidity sensing [59, 87].

Post‐traumatic inflammation and gliosis induce localized tissue stiffening and transmit persistent mechanical stress to the PSD through the integrin axis [30, 77, 82, 85]. ECM remodeling establishes mechanical memory that imposes non‐physiological tension on the neuronal interior [30, 77, 78, 79]. Under this feedback, dendritic spine morphodynamics are constrained, and a rigid substrate limits PSD condensate expansion, suppressing synaptic potentiation and structural remodeling [82, 85]. Because brain tissue is viscoelastic, impact loading generates shear strain and tensile stress [8, 15, 88]. Mechanical energy concentrates at interfaces with mismatched elastic moduli, especially in anisotropic structures such as white matter tracts and dendritic branches [10, 58, 85, 89, 90].

In this context, the dense PSD behaves as a relatively stiff domain within a softer cellular matrix. When external stress is perpendicular to the neuronal axis, shear forces on the synaptic cleft expose the PSD periphery and cytoplasmic interface to abnormal loads [8, 15, 85]. Synaptic plasticity also depends on Shank3‐mediated PSD stiffening; impact‐related disruption of Shank3 oligomerization reduces PSD stiffness and accelerates LTP decay [47].

### 3.2 PSD as a Load‐Bearing Axis

When mechanical energy converges from the ECM to the synapse, the PSD shifts from a signaling platform into a load‐bearing scaffold axis. Extreme strain distorts or ruptures the scaffold network, weakening the physical interactions that maintain condensate toughness. Post‐impact PSD perforation or fragmentation reflects physical disassembly of the scaffold hierarchy [22, 29, 54, 55]. These scaffold proteins not only anchor receptors but also define PSD material properties and transmit mechanical signals [49, 91]. By altering receptor conformations and protein coupling, mechanical stress links microscopic displacement to pathological biochemical cascades.

#### 3.2.1 Receptor Mechanogating and Piezo1‐Mediated Influx

At the synaptic entry point, mechanical force directly modulates receptor state. NMDARs exhibit mechanogating, in which membrane stretch mimics ligand‐induced conformational changes and opens ion channels without glutamate [70, 82]. This produces a non‐physiological ionic surge and rapidly initiates excitotoxic programs [8, 70]. Preso facilitates the mGluR1–Homer1 complex and interacts with PSD‐95 and CDK5 to amplify calcium and nitric oxide signaling [37, 92]. Extreme stretching distorts these coupling interfaces, promotes lateral diffusion of anchored receptors such as AMPARs, and impairs synaptic transmission [15, 22, 85].

Among mechanosensitive channels, Piezo1 is a key entry point for physical impact because of its high sensitivity [71, 72, 73]. Its transmembrane blades and intracellular beam detect millisecond‐scale membrane tension, and increased tension drives channel opening through blade displacement [71, 74]. Under TBI loading, Piezo1 activation causes transient Na^+^ and Ca^2+^ influx, local calcium surges, and rapid postsynaptic depolarization before NMDAR activation [15, 74, 75]. Piezo1 inhibition attenuates calcium overload and PSD‐95 loss, supporting its upstream role in the mechanical response to TBI [55, 75]. Piezo1 sensitivity is further enhanced by TBI‐induced disruption of actin dynamics [93], whereas post‐traumatic gliosis can produce aberrant Piezo1‐mediated feedback that affects long‐term PSD stability [73, 94]. Piezo1 activation also modulates post‐injury cytokine release [15, 85].

Mechanosensitive responses vary by region. Hippocampal neurons show greater intracellular calcium fluctuations than cortical neurons during injury [15, 57]. This difference reflects regional tissue moduli and strain‐energy unloading [57, 58]. Initial ion influx through mechanosensitive channels can then alter mitochondrial membrane permeability, contributing to ATP depletion and apoptosis [14, 76, 95, 96].

#### 3.2.2 Scaffold Decoupling and Conformational Pathology

Within the PSD, mechanical signals propagate along the membrane receptor–PSD‐95–SAPAP–Shank–actin axis [24, 43, 85]. TBI decouples this axis from the receptor layer to the cytoskeleton. Piezo1, which is normally tethered to the actin cytoskeleton through the cadherin–β‐catenin mechanotransduction complex, is susceptible to failure under shear stress [85, 93]. This failure is amplified by calcium overload, which mimics plasticity signals, promotes abnormal degradation of SynGAP1 and PSD‐95, and drives a maladaptive synaptic state [15, 22, 42, 74].

When impact forces exceed the critical stress of the PSD condensate—a failure threshold that has not yet been measured and is used here qualitatively—internal protein bonds may rupture, and the PSD may shift from an ordered condensed state to a diffuse state [6, 47]. This structural disintegration reduces the mechanical modulus required for long‐term potentiation, providing a biomechanical basis for post‐TBI memory impairment [23, 38]. Compensatory PSD‐95 increases after injury do not necessarily ensure neuroprotection [38, 97, 98], and PSD‐95 overexpression can exacerbate NMDAR‐mediated excitotoxicity [99]. In a mechanically damaged PSD, repair fails if the scaffold hierarchy, especially the SAPAP/Shank axis, remains disrupted [28, 43].

Extreme strain stretches scaffold proteins and converts mechanical load into biochemical damage [4, 15, 47]. This deformation exposes cryptic cleavage sites, allowing PSD‐localized calpain to degrade PSD‐95, Shank3, and SynGAP1 [15, 22, 42, 47]. After mechanosensitive receptor opening, calcium‐dependent signaling is amplified through CaMKII, Src family kinases, and GSK3β. Force‐induced calcium surges through mechanosensitive channels can trigger CaMKII autophosphorylation at Thr286, which locks the kinase in a persistently active conformation [100, 101]. Activated CaMKII hyperphosphorylates SynGAP1 and forces its desorption from the phase‐separated condensate, driving downstream signaling disorder and postsynaptic hyperexcitability [41, 42]. Beyond SynGAP1, CaMKII also phosphorylates SAPAP within its guanylate‐kinase‐binding repeats, which markedly strengthens SAPAP–PSD‐95 binding and drives the fusion of upper‐ and lower‐layer PSD condensates into a single assembly, a process reversed by dephosphorylation [102]. In addition, CaMKII itself acts as an organizational component of the PSD: in its autoinhibited state it is recruited into the Shank3 subcompartment through phase separation, and Ca^2+^‐dependent, phosphatase‐checked shuttling of CaMKII between Shank3‐ and GluN2B/PSD‐95‐containing nanodomains couples kinase activation to PSD assembly and structural synaptic plasticity [103]. These additional functions position CaMKII as a multifaceted regulator of PSD material state and plasticity after mechanical injury. Strain‐induced Src activation phosphorylates GluN2B and enhances NMDAR excitotoxicity [8, 15, 104]. GSK3β‐mediated phosphorylation destabilizes the PSD‐95/APC/EB3 complex and decouples the scaffold from the cytoskeleton [38]. These cascades convert transient displacement into chronic architectural disruption [47, 85].

#### 3.2.3 Hub‐Centered Network Vulnerability

The PSD can be conceptualized as a hub‐centered scaffold network in which highly connected components such as PSD‐95 and Shank3 create mechanical vulnerability. Failure of these nodes can trigger cascading topological disruption and leave neurons molecularly disconnected [55, 105]. Based on this view, we propose the sacrificial protection hypothesis: PSD protein interactions may act as synaptic mechanical fuses, absorbing energy through partial disassembly and protecting the postsynaptic membrane from rupture [15]. The cost is long‐term synaptic dysfunction.

Direct data defining scaffold fracture sequences under mechanical loading remain scarce. Single‐molecule force spectroscopy, including atomic force microscopy and optical tweezers, provides in vitro stability parameters [62], but has not been integrated with brain tissue or TBI models. Future studies should determine fracture thresholds, disassembly sequences, and repair dynamics of PSD scaffold proteins to link mechanical failure with synaptic dysfunction after TBI.

### 3.3 Astrocytic Metabolic and Immune Relay

Synaptic mechanical homeostasis is not maintained by neurons alone. As components of the tripartite synapse, astrocytes use mechanosensing pathways to regulate PSD responses after TBI [22, 80, 105, 106]. Astrocytic Piezo1 channels sense stress fluctuations and trigger ATP release; extracellular ATP then acts on neuronal P2X receptors, forming a mechano‐electro‐chemical relay that modulates synaptic weights and PSD component organization [105]. This relay converts transient deformation into chemical signaling that alters postsynaptic excitability.

Mechanical strain also promotes a local metabolic crisis. Altered astrocyte membrane tension inhibits glutamate transporter activity, delays neurotransmitter clearance, and prolongs NMDAR activation [15, 107]. Intense loading also changes glial mitochondrial membrane permeability, causing ATP depletion and metabolic imbalance [14, 76]. Energy failure then restricts active transport and dynamic exchange required for PSD maintenance.

Over longer timescales, astrocytes contribute to immune‐mediated PSD remodeling. During the subacute phase, especially 3–7 days after injury, PSD degradation and astrocyte phagocytosis occur in hippocampal CA1 [108]. Aquaporin‐4 (AQP4) helps clear PSD degradation products through the glymphatic system, but in human blast‐induced TBI and mouse models, AQP4 expression increases while perivascular polarity decreases, potentially impairing synaptic debris clearance [109]. Astrocytic Piezo1 also links ion flux with immune feedback that suppresses inflammatory cytokines [15, 85]. When damage is irreversible, astrocytes adjust synaptic coverage and use D‐serine and complement pathways to prune damaged PSDs [22, 80, 105]. Thus, astrocytes provide a metabolic and immune relay that shapes PSD structural fate after mechanical perturbation (Figure 2).

**FIGURE 2:**
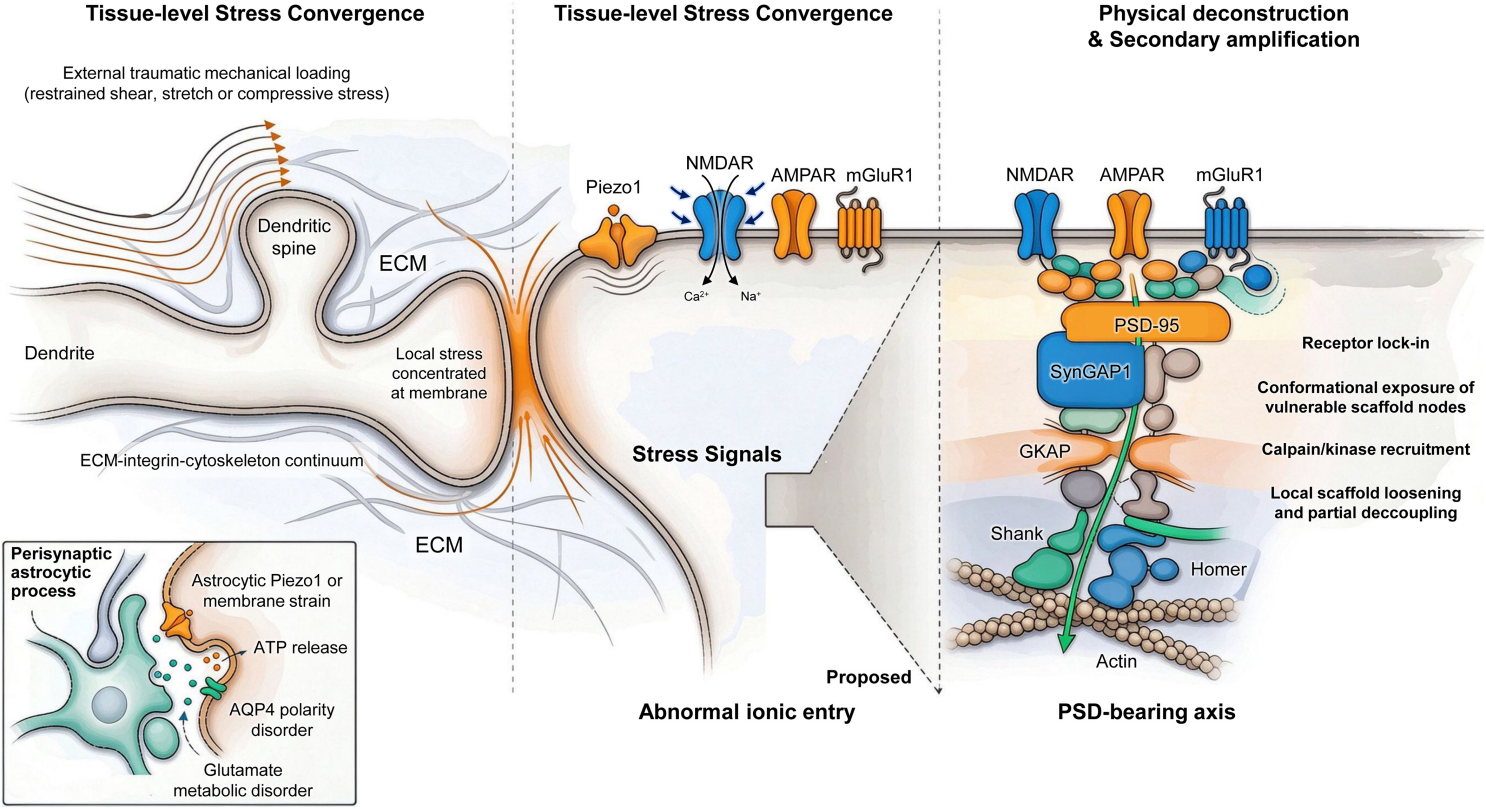
Sequential force relay from tissue stress convergence to destabilization of the PSD load‐bearing axis. Traumatic mechanical loading (shear, stretch, or compression) converges through the extracellular matrix (ECM), integrin‐linked membrane interfaces, and the cytoskeletal continuum toward the synaptic boundary. At the postsynaptic membrane, mechanical perturbation activates mechanosensitive entry points, including Piezo1, and promotes abnormal receptor gating, especially at NMDARs, leading to excessive cation influx (Ca^2+^, Na^+^). Beneath this interface, the PSD load‐bearing axis (PSD‐95/SynGAP1 hub, SAPAP/GKAP bridging region, and Shank/Homer/Actin scaffold) becomes strained, misaligned, and destabilized, with conformational exposure of vulnerable scaffold nodes and calpain/kinase recruitment. The inset illustrates astrocyte‐mediated amplification through mechanosensing, ATP release, AQP4 polarity loss, and impaired glutamate clearance. Evidence key: Solid lines denote established pathways supported by the cited evidence; dashed lines denote proposed force‐transmission steps and the astrocytic Piezo1–ATP relay inferred from mechanotransduction studies in non‐TBI systems [71, 72, 73, 74, 105].

## 4 Temporal Pathological Evolution of the PSD: From Instantaneous Physical Perturbation to Chronic Structural Consolidation

TBI evolves across timescales from milliseconds to decades [4, 9, 30], and PSD disruption follows a similar continuum from primary mechanical injury to secondary cascades [8, 15]. Neuropathological evidence further frames TBI as a multi‐morbidity with lifelong health effects [9, 110]. At impact, tissue displacement and deformation injure the neuronal microenvironment [10, 11, 111, 112], whereas mechanosensitive channels open and drive rapid ionic influx at the synapse [70, 71]. The acute phase is dominated by calcium‐dependent protease activation and cytoskeletal reorganization [8, 15], together with metabolic dysfunction, mitochondrial failure, and inflammatory edema [11, 12, 13, 14]. In the chronic phase, neuroinflammation and oxidative stress contribute to structural consolidation [16, 22, 29, 113] and increase the risk of CTE, epilepsy, and dementia [5, 110, 114, 115, 116]. The PSD may therefore serve as a molecular site where transient physical forces are converted into long‐term synaptic pathology [2, 5, 22, 23, 29, 38] (Figure 3).

**FIGURE 3:**
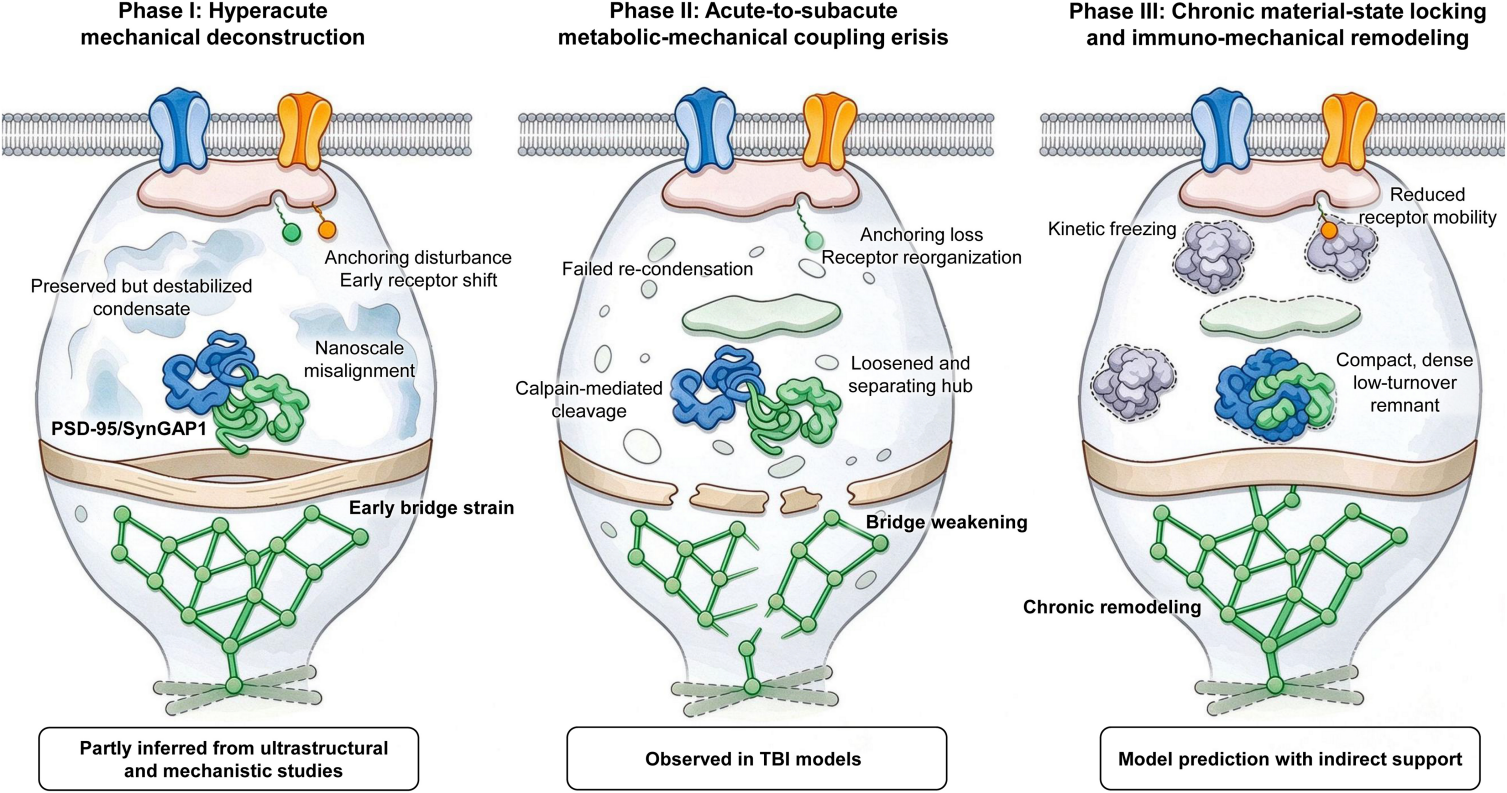
Time‐resolved mechanical evolution of the postsynaptic density (PSD) after traumatic loading. Three phase‐specific state portraits compare the same membrane‐associated PSD condensate across pathological stages. Phase I (hyperacute, milliseconds to minutes): The PSD remains largely preserved but shows early phase loosening, nanoscale misalignment, slight receptor shift, initial hub displacement, and SAPAP‐linked bridge strain. Phase II (acute‐to‐subacute, minutes to weeks): Mechanical perturbation couples with metabolic and proteolytic stress, and the condensate becomes diffuse and less cohesive, with calpain‐mediated cleavage, receptor reorganization, hub loosening, bridge weakening, and failed re‐condensation. Phase III (chronic, months to years): The PSD adopts a pathologically stabilized low‐dynamic state with kinetic freezing, reduced receptor mobility, low‐turnover scaffold remnants, and a stiffened deep architecture, reflecting pathological locking and persistent remodeling. Evidence status (labeled on the figure): Phase II changes are observed in TBI models [66, 108, 117, 118, 119, 120, 121, 122, 123, 124]; Phase I nanoscale deconstruction is inferred from ultrastructural and mechanistic studies and remains partly hypothetical; Phase III pathological locking is a model prediction supported indirectly by chronic molecular and ECM changes [30, 77, 79, 123, 124, 125, 126, 127, 128].

### 4.1 Phase I: Hyperacute Mechanical Deconstruction

On the millisecond‐to‐minute timescale, the PSD undergoes hyperacute mechanical deconstruction. This phase is defined by physical decoupling under extreme strain before full biochemical degradation begins. Brain tissue anisotropy creates non‐uniform stress at the synaptic interface and generates localized shear forces [15, 89]. Under high strain rates, non‐covalent bonds that stabilize the PSD scaffold, including van der Waals forces, hydrophobic interactions, and multivalent electrostatic attractions, undergo physical rupture [4, 54].

This damage appears as a mechanically induced phase transition, collapsing high‐stiffness PSD condensates into a loose, disordered, or diffuse state [47, 55, 56]. At this stage, PSD changes mainly reflect topological disorganization rather than immediate protein loss. PSD‐95 and Shank3 may undergo pathological displacement from the central postsynaptic membrane toward the periphery [22, 85]. This positional pathology disrupts nanoscale alignment between postsynaptic receptors and the presynaptic active zone, impairing synaptic transmission [15, 39].

Rapid molecular changes support this phase. Homer1 levels decrease as early as 5 min after injury [129]. By 15 min, hippocampal fractions show increased NMDAR subunits (GluN1, GluN2A/B) and AMPAR GluA1, together with elevated Tyr1472 phosphorylation of GluN2B and ERK activity, indicating early compromise of synaptic architecture [130].

Two testable predictions follow for this phase. First, PSD‐95 and SynGAP1 mobility and the nanoscale alignment of receptors with presynaptic release sites should change within milliseconds to minutes of loading, before any loss of total protein is detectable. Second, these changes should scale with the applied strain rate. Observing stable scaffold mobility and preserved alignment under loading conditions that nevertheless produce synaptic dysfunction would disconfirm mechanical deconstruction as an initiating event.

### 4.2 Phase II: Metabolic–Mechanical Crisis in the Acute‐to‐Subacute Phase

In this phase, hyperacute physical damage is consolidated by calcium overload, energy failure, and protease activation. Mechanical strain exposes cryptic sites in loosened scaffold proteins, and post‐TBI calcium surges activate PSD‐localized calpain, which cleaves PSD‐95, Shank3, and SAPAP [22, 38, 47, 82]. This converts potentially reversible physical decoupling into biochemical degradation.

During the acute phase, especially 24–72 h after injury, core scaffold proteins undergo time‐dependent degradation. PSD‐95 levels decrease, with low‐molecular‐weight fragments appearing despite stable mRNA levels, indicating protein degradation rather than transcriptional downregulation [66, 108]. NMDAR subunits (GluN1, GluN2A/B) decrease between 6 and 12 h [117], accompanied by impaired receptor anchoring marked by reduced p‐Stargazin and p‐GluA1 [118, 119]. Arc peaks at 12 h as a stress‐responsive regulator of AMPAR trafficking, and its knockdown worsens cytotoxicity and RIP1‐dependent necroptosis [120, 121].

By 3–7 days, PSD remodeling becomes region‐specific. The cortex may show compensatory increases in PSD thickness, whereas hippocampal CA1 shows reactive regeneration with reduced active zone length [108, 122]. Homer1 remains below baseline by day seven despite spine‐head enlargement [129]. Tau and Aβ accumulation during this window links acute synaptic structural damage to later proteinopathy [110, 116]. Within 1–2 weeks, total and phosphorylated Neurogranin remain reduced in injured hippocampus and cortex, indicating persistent mismatch between structural and functional synaptic components [123, 124].

This phase is also metabolic. PSD maintenance depends on LLPS‐mediated component exchange, but post‐injury ATP deficits caused by energy depletion and astrocytic dysfunction impair this process [14, 76]. Without sufficient ATP, dispersed scaffold proteins fail to re‐condense, producing diffuse, perforated, or sparse PSD structures [4, 76]. Aberrant kinase activity, including GSK3β‐related phosphorylation, further weakens scaffold affinity for the actin cytoskeleton [22, 46]. This loss of mechanical anchoring defines the metabolic–mechanical crisis: biochemical cascades damage PSD proteins and the physical conditions required for material repair [22].

Morphological data support this model. In a rat hippocampal ischemia–reperfusion model, CA1 PSD thickness increased from 49.8 to 88–95 nm, PSD area from 4.0 × 10^3^ nm^2^ to 7.5–8.3 × 10^3^ nm^2^, and perforation rate from 4.3% to 6.6%, whereas DG changes were smaller and transient. Three‐dimensional reconstructions showed irregular, loosened PSDs, supporting region‐specific and time‐dependent structural remodeling after acute injury [125]. Chronic TBI‐specific quantitative data on PSD thickness, area, and ultrastructure remain limited.

This phase makes two testable predictions. First, the re‐condensation of dispersed scaffold proteins should depend on ATP availability, so restoring ATP or blocking calpain within the first 24–72 h should preserve PSD integrity. Second, condensate material properties should change before gross ultrastructural perforation becomes detectable. Failure of ATP restoration or calpain inhibition to preserve PSD structure would argue against a metabolic–mechanical crisis as defined here.

### 4.3 Phase III: Low‐Dynamic Pathological Locking and Immuno‐Mechanical Remodeling

In the chronic phase, the initial mechanical impulse can persist as mechanical memory. Chronic neuroinflammation drives extracellular matrix (ECM) remodeling, including hyaluronic acid and chondroitin sulfate proteoglycan deposition, increasing peri‐synaptic stiffness [30, 77, 79]. This modulus mismatch transmits non‐physiological tension through the integrin–ROCK axis, enhances actin contractile stress, and anchors PSD scaffold proteins in a high‐tension, low‐dynamic state [72, 85]. We define this process as low‐dynamic pathological locking, in which the PSD loses the molecular exchange required for synaptic plasticity [55, 105]. “Low‐dynamic pathological locking” is used as the preferred term for this chronic material state throughout this review; “solidified” and “kinetic freezing” denote the same state and are used only descriptively.

Chronic PSD remodeling shows fluctuating molecular changes. At 4 weeks post‐injury, hippocampal PSD‐95 may show a super‐compensatory increase, whereas p‐neurogranin remains elevated [123, 124]. By 5 weeks, repetitive mild TBI increases cortical calpain‐1, p‐GluR1, and amyloid precursor protein (APP) [126]. At 12 weeks, GluN1 increases in the prefrontal cortex and amygdala and correlates with delayed‐onset depression [127]. At 32 days, PSD‐95 intensity decreases in regions including CA1 and the nucleus accumbens, whereas astrocytic NLRP3‐cKO enhances PSD‐95 intensity and improves function, suggesting neuroinflammatory regulation of chronic synaptic damage [128].

Microglia further shape chronic immuno‐mechanical remodeling. Unrepaired cryptic sites exposed by initial mechanical stress can be recognized through complement pathways, including C1q and C3 [22, 80]. Activated microglia clear residual PSD fragments, but excessive pruning may damage preserved synaptic connections. Stretch injury increases Iba‐1 levels and microglial migratory persistence [81], and diffuse mechanical injury signals may convert early perturbation into long‐term circuit disintegration [80, 131].

Finally, degradation products and abnormal Tau deposition increase molecular crowding within the PSD [55]. This can induce kinetic freezing, shifting the PSD from a dynamic LLPS state to a solid‐like, poorly responsive structure [47, 55]. In this low‐dynamic pathological locking state, receptor lateral diffusion is restricted, limiting postsynaptic adaptation even after partial recovery of presynaptic release. This may explain persistent dysfunction in structurally preserved synapses [105] (Table 2). Future studies should quantify LLPS reversibility, receptor diffusion, re‐condensation kinetics, and molecular crowding under chronic mechanical load.

**TABLE 2:** PSD stress responses across temporal scales.

Component	Phase I: Hyperacute phase (ms–min)	Phase II: Acute‐to‐subacute phase (min–weeks)	Phase III: Chronic phase (months–years)
Membrane receptors (NMDAR/AMPAR)	Mechanogated activation: Membrane tension induces conformational changes and ligand‐independent channel opening [70, 82]; spatial misalignment: Receptors undergo pathological lateral diffusion toward the synaptic periphery [15, 22, 85]	Subunit reorganization: Receptor composition changes under sustained excitotoxic stress [21]. Receptor abundance decreases as scaffold degradation impairs anchoring [22, 38]	Kinetic Freezing: Receptor lateral diffusion is restricted in a crowded PSD environment, limiting rearrangement during LTP and other plasticity signals [55, 105]
Core scaffold hubs (PSD‐95/SynGAP1)	Physical displacement: Components are not immediately lost but show nanoscale positional deviation under high strain rates, producing position pathology [22, 85]	Biochemical degradation: Calpain cleaves proteins with exposed cryptic sites. Slot competition loss: SynGAP1 depletion disrupts spatial organization within the PSD [22, 38, 41, 42]	Structural degeneration: Residual proteins fragment or form nonfunctional aggregates, whereas scaffold turnover remains chronically low under energy deficiency [76, 105]
Deep scaffold axis (Shank3/SAPAP)	Oligomeric network rupture: Physical bonds between SAM domains break under critical stress [47]; damping failure: SAPAP loses its capacity to buffer instantaneous mechanical impulses [45]	Scaffold decoupling: GSK3β‐Related phosphorylation weakens anchoring between deep scaffold proteins and the actin cytoskeleton [22, 46]	Pathological locking: ROCK‐axis activation fixes components in a high‐tension, low‐dynamic state, reducing LLPS‐driven reorganization [72, 85]
Microenvironment and ECM	Stress convergence: Mechanical loading generates shear strain at interfaces with synaptic modulus mismatch [8, 15]; glial sensing: Piezo1 responds to membrane tension [105]	Metabolic exhaustion: ATP depletion disrupts LLPS dynamics. Relay crisis: impaired astrocytic glutamate transport amplifies NMDAR‐mediated damage [14, 15, 76, 107]	Mechanical memory: Persistent ECM stiffening generates chronic static tension [30, 77, 79]; excessive immune pruning: Microglia remove residual fragments in response to injury‐associated cues [80]

*Note:* Operational definitions: “high strain rate,” loading faster than approximately 1 s^−1^; “critical stress,” the not‐yet‐determined failure threshold of PSD condensate integrity; “tissue stiffness,” the shear or elastic modulus of brain tissue (approximately 0.5–2 kPa [58]); “membrane tension,” the in‐plane bilayer tension gating mechanosensitive channels (Piezo1 half‐maximal activation at approximately 1.4–2.7 mN/m [60]); “mechanical locking,” kinetic suppression of molecular exchange within the PSD (a model prediction, not a measured phase). Evidence levels for the processes summarized here are assigned in Table 1. Timescales are approximate and vary by species and injury model.

This phase predicts that softening the ECM or inhibiting the integrin–ROCK axis should restore PSD component exchange and receptor mobility even at chronic time points. Persistence of a low‐dynamic, locked state despite such manipulation would falsify the mechanical‐memory account.

### 4.4 Testable Predictions and Falsifiability of the Temporal–Mechanical Model

For this framework to be useful, it must be falsifiable. Building on the phase‐level predictions noted above, we specify measurable readouts and disconfirming outcomes for each phase (Table 3). Phase I predicts that PSD‐95/SynGAP1 mobility and exchange, measured by single‐molecule tracking or fluorescence recovery after photobleaching in stretch‐injured neurons, decrease or redistribute within milliseconds to minutes and before detectable protein loss, and that single‐synapse receptor nanodomain alignment degrades within the same window. Preserved mobility and alignment under dysfunction‐producing loads would disconfirm this step. Phase II predicts that re‐condensation of dispersed scaffolds is ATP‐dependent and calpain‐sensitive within hours of injury, and that condensate material properties, such as viscosity and molecular exchange rate, change before ultrastructural perforation. If ATP restoration or calpain blockade fails to preserve PSD integrity, or if proteolysis alone fully accounts for scaffold dispersion without any ATP‐sensitive component, the crisis concept would be weakened. Phase III predicts that chronically injured tissue combines increased ECM stiffness with reduced PSD component exchange, and that ECM softening or integrin–ROCK inhibition restores PSD turnover and receptor mobility within days. Persistent dysfunction without ECM stiffening or without reduced exchange, or failure of mechanical rescue to restore plasticity, would require the locking model to be revised or abandoned.

**TABLE 3:** Falsifiable predictions of the temporal–mechanical model by phase.

Phase (temporal window)	Measurable prediction	Disconfirming outcome	Distinction from alternative models
Phase I: hyperacute (milliseconds–60 min)	FRAP or single‐particle tracking shows reduced PSD‐95/SynGAP1 mobility and exchange within minutes of injury, before any loss of total protein	Mobility changes appear only after, or simultaneously with, protein degradation	Supports mechanical deconstruction rather than proteolysis‐first models
Phase I: hyperacute (min–h)	Super‐resolution imaging shows nanoscale receptor–active‐zone misalignment at synapses retaining normal protein abundance	Misalignment occurs only where protein loss has already occurred	Supports position pathology rather than excitotoxicity‐driven loss
Phase II: acute–subacute (6 h–7 days)	Restoring ATP availability or blocking calpain rescues PSD re‐condensation and condensate material properties in injured tissue	Re‐condensation proceeds normally despite ATP depletion, or calpain blockade fails to preserve condensate state	Supports a metabolic–mechanical crisis rather than a purely excitotoxic cascade
Phase II: acute–subacute (24 h–2 weeks)	Condensate material properties (viscosity, exchange rates) in injured synapses diverge from protein‐abundance measures	Material measures track protein abundance one‐to‐one	Supports a material‐state transition rather than quantitative component loss
Phase III: chronic (2–12 weeks)	FRAP shows chronically suppressed PSD molecular exchange co‐localized with stiffened ECM; normalizing substrate stiffness or integrin–ROCK signaling restores exchange	Exchange normalizes spontaneously despite persistent ECM stiffening, or stiffness normalization has no effect	Supports mechanical locking rather than conventional plasticity failure
Phase III: chronic (days–months)	Microglial engulfment of PSD material is gated by mechanical cues (cryptic‐epitope exposure or complement deposition on strained synapses)	Pruning rate is independent of the local mechanical state	Supports immuno‐mechanical remodeling rather than activity‐dependent pruning alone

Each prediction carries a temporal window so that the temporal–mechanical model can be distinguished from alternative accounts. Pure excitotoxicity models predict calcium‐dependent damage that does not scale with strain rate; proteolysis‐only accounts predict irreversible scaffold loss rather than initially reversible dispersion; and conventional synaptic plasticity models do not predict material‐state transitions of the PSD condensate. Live measures of PSD‐95/SynGAP1 mobility, single‐synapse receptor nanodomain alignment, condensate material properties, ATP dependence, ECM stiffness, and microglial engulfment therefore constitute a concrete experimental program for confirming or refuting the framework.

## 5 Conclusion and Outlook: Toward Mechano‐Informed Therapeutic Hypotheses

TBI evolves from physical impact to biochemical and structural remodeling, with long‐term effects on morbidity and mortality [2, 5, 8, 29]. In this process, the PSD acts as both a mechanical buffer and a biochemical relay [22, 23, 38]. Mechanical strain disrupts the PSD scaffold network, and later remodeling impairs synaptic transmission and plasticity, including LTP/LTD [22, 25]. These changes support a temporal–mechanical model in which PSD injury progresses from hyperacute mechanical deconstruction to metabolic–mechanical crisis and low‐dynamic pathological locking.

Mechanobiology links tissue‐level force to synaptic dysfunction. Post‐injury inflammation and glial proliferation increase ECM stiffness, restrict PSD condensate expansion, and suppress synaptic potentiation by altering molecular crowding and receptor mobility [82, 85, 132, 133]. However, cross‐temporal PSD studies remain limited, partly because TBI model heterogeneity complicates comparison across injury profiles [2, 15, 30] (Table 4). Future work should quantify strain thresholds across tissue components and integrate finite element brain models with high‐resolution transcriptomics to improve injury assessment and therapeutic stratification [61, 64, 65].

**TABLE 4:** PSD component changes across diverse TBI models and associated limitations.

Component	Species	Model	Main changes	Limitations	References
PSD‐95	Rat	CCI	Contusional cortex: PSD‐95 and synaptophysin decrease over 1–7 days in association with oxidative stress	Cortex only; total protein by Western blot	[67]
	Rat	Marmarou weight‐drop	Hippocampal PSD‐95 and synaptophysin significantly reduced at 3 and 5 days; resveratrol partially reverses the decrease	Hippocampus only; pharmacological reversal is associative	[68]
	Rat	PBBI	24 h: main band reduced by ~69% ipsilaterally with 38/25 kDa fragments; DISC1 and NOS1 reduced; mRNA unchanged, indicating protein degradation rather than transcriptional downregulation	Penetrating model; fragments not sequenced	[66]
	Rat (organotypic hippocampal slices)	Primary blast (in vitro)	PSD‐95 reduced after blast; LTP impaired within hours and recovering by 10 days; PDE4 inhibition restores LTP and ameliorates protein changes	In vitro preparation; not an in vivo blast model	[118, 119]
	Mouse	CCI	Delayed hippocampal decrease over 1–7 days, temporally correlated with cognitive dysfunction	Quantitative effect size not consistently reported	[69]
Neurogranin	Mouse	TBI	7 days: PSD‐95 and synapsin‐1 reduced, with reduced dendritic branching and spine density; YL‐0919 reverses these changes	Pharmacological reversal is associative	[134]
	Mouse	rmTBI (closed‐head)	No significant change at 3 days or 5 weeks	Negative finding; few time points	[126]
	Rat	CCI	Total and phosphorylated neurogranin reduced in injured cortex and hippocampus from 24 h to 2 weeks; sex‐dependent trajectories over 1–4 weeks (greater loss in females; biphasic p‐Ng/Ng ratio)	Sex‐by‐time interactions; region‐specific assays	[123, 124]
NMDAR subunits	Rat	CCI	Hippocampal NR1, NR2A and NR2B protein transiently reduced at 6–12 h and fully recovered by 24 h; mRNA unchanged	Hippocampus only; transient window	[117]
	Rat	Closed‐head CCI	12 weeks: GluN1 increased in frontal cortex and amygdala, correlating with depression‐like behavior; hippocampal GLT‐1 reduced	Male rats only; behavioral correlation is associative	[127]
	Mouse	Closed‐head injury (weight‐drop)	15 min: hippocampal NR1, NR2A, NR2B and p‐NR2B (Tyr1472) increased with elevated ERK activity; opposite direction at the impact‐site cortex	Single hyperacute time point; regional divergence	[130]
	Mouse	rmTBI (closed‐head)	3 days: cortical NR2B reduced (NR1/NR2A unchanged) with increased p‐CaMKII; NR2B recovers by 5 weeks; environmental enrichment mitigates deficits	Phosphorylation readouts predominate	[126]
AMPAR (GluR1/GluA1)	Mouse	Closed‐head injury (weight‐drop)	15 min: hippocampal GluR1 transiently increased	Single hyperacute time point	[130]
	Mouse	rmTBI (closed‐head)	5 weeks: cortical p‐GluR1 increased while total GluR1 is unchanged	Phosphorylation site not linked to behavior	[126]
	Rat (organotypic hippocampal slices)	Primary blast (in vitro)	Total GluR1 and p‐GluR1 (Ser831) reduced, with reduced p‐stargazin (Ser239/240); PDE4 inhibition ameliorates the changes	In vitro preparation	[118, 119]
Homer1	Mouse	mTBI (closed‐head)	Homer1‐positive puncta show a downward trend at 5 min (not significant) and are significantly reduced at 7 days in BBB‐disrupted regions; the phenotype persists up to 6 months without neuronal death	Puncta counts, not total protein; restricted to BBB‐disrupted zones	[129]
	Rat	CCI	No significant change during the acute phase (3–72 h)	Negative finding; few time points	[121]
Arc	Rat	CCI/in vitro traumatic neuronal injury	Stress‐responsive upregulation peaking at ~3–12 h and returning to baseline by 24 h; Arc silencing aggravates mGluR1‐mediated ER stress and necroptosis	Peak timing varies across models; functional role inferred from silencing	[120, 121]
CaMKII	Rat	CCI	Neurogranin/CaMKII signaling shows sex‐ and time‐dependent changes over 1–4 weeks	Phosphorylation readouts; sex‐dependent trajectories	[124]
	Mouse	rmTBI (closed‐head)	p‐CaMKII increased in cortex and hippocampus at 3 days	Phosphorylation readout only	[126]

This framework also requires experimental validation. Current evidence comes from TBI models, related CNS injury paradigms, mechanotransduction studies, and condensate biology. PSD scaffold remodeling, receptor mobility, and condensate‐state changes should be tested across standardized TBI models, brain regions, injury severities, and time windows. Such studies should determine whether PSD changes are causal drivers, adaptive responses, or secondary consequences of post‐traumatic synaptic dysfunction.

Therapeutically, these ideas remain candidate hypotheses rather than near‐term treatments. If PSD material state contributes to post‐TBI dysfunction, strategies that stabilize membrane tension, modulate Piezo1 gating, or tune peri‐synaptic tissue stiffness with biomaterials could be explored [55, 73, 75]. Stabilizing Shank3 oligomerization or modulating SAPAP phosphorylation might, in principle, restore PSD material properties [43, 47], and astrocytic Piezo1 signaling links scaffold regulation to tissue mechanics [4, 105, 114]. A complementary strategy is to rebuild trans‐synaptic connectivity directly: the synthetic synaptic organizer CPTX, which bridges neurexin and AMPA receptors, restores excitatory synaptic transmission and circuit function in mouse models of cerebellar ataxia, Alzheimer's disease, and spinal cord injury [135], illustrating how structural synapse organizers might be adapted to post‐traumatic synapse loss.

All of these directions face substantial translational barriers: delivering macromolecules or biomaterials across the blood–brain barrier; achieving cell‐type and synapse specificity; defining the therapeutic window across the hyperacute, acute, and chronic phases; avoiding off‐target effects; and, critically, preserving the physiological mechanotransduction that normal synapses require for plasticity. No validated clinical endpoints currently exist for PSD‐targeted or mechanics‐targeted therapies, and none of these approaches has been tested in human TBI. We therefore present “mechano‐therapeutics” as a research agenda—a set of testable, mechanics‐informed hypotheses whose value will be determined by the falsification studies outlined above.

## Author Contributions

Yibin Jia and Zixuan Cao wrote the manuscript, Yibin Jia and Hanjiang Xue designed and produced the figure and the table. Zhebin Feng and Zhiguo Ma conducted literature searches and induction. Zhicheng Tian, Xin Li, and Peng Luo outlined, supervised, and edited the draft and approved the final version to be submitted. All authors approved the final version of the manuscript.

## Funding

We would like to express our sincere gratitude to the funding support provided by the National Natural Science Foundation of China (Nos. 82571563, 82371381, and 82171363 for Peng Luo; 82571666 for Xin Li). This work was also supported by the Hospital Project of PLA 942 Hospital (L942Y2025007) to Yibin Jia.

## Ethics Statement

The authors have nothing to report.

## Conflicts of Interest

The authors declare no conflicts of interest.

## Data Availability

Data sharing not applicable to this article as no datasets were generated or analyzed during the current study.
